# Only-Glue Fixation in Laparoscopic Intraperitoneal Onlay Mesh Repair of Midline Ventral Hernias: Technical Aspects and Short-Term Results of 40 Patients

**DOI:** 10.7759/cureus.85324

**Published:** 2025-06-04

**Authors:** Joaquin Picazo-Yeste, Syed A Faheem, Khadiga Abdelmonem, Bipin P Thomas, Saad Elhanafy, Mohammed A El Gazzar, Nisreen Al Aamri

**Affiliations:** 1 General Surgery, Burjeel Medical City, Abu Dhabi, ARE; 2 Medicine, Burjeel Medical City, Abu Dhabi, ARE

**Keywords:** cyanoacrylate glue, hernia, laparoscopic ipom repair, mesh fixation, tissue sealant

## Abstract

Mesh fixation remains a key challenge in laparoscopic hernia repair, with no universally accepted technique established to date. This study investigates the clinical application of cyanoacrylate (CA) as the sole fixation method in laparoscopic intraperitoneal onlay mesh (IPOM) repair for midline ventral hernias.

A retrospective analysis of patients with midline ventral hernias who underwent IPOM-only glue (IPOM-OG) repair from September 2022 to December 2023 has been carried out. Follow-up was available in 34 (85%) of 40 patients who were operated on during the study period. The majority were primary umbilical hernias, with an average width of 3 cm. Closure of the defect (IPOM +) was performed in 25 (74%) cases, the average hernia-mesh size ratio was 1:16, and the operative time was 64 (40-90) minutes. Two (6%) pseudo-hernias (seroma at the residual sac) were successfully treated with fine-needle percutaneous aspiration. Postoperative visual analog scale (VAS) score of pain in two different measures was low, and no patient complained of chronic pain. Two patients underwent another laparoscopic procedure (cholecystectomy) 14 and 90 days after IPOM-OG, respectively. In both cases, we could confirm that the mesh was adequately positioned without disruptions, wrinkles, bulges, or curled edges. After a median follow-up of 16 (12-26) months, no recurrences or abdominal bulging have been detected.

Our proposed technique provides additional data regarding the use of CA as a sole fixation method in IPOM repair of midline ventral hernias. It resulted in a reduction of chronic pain, without jeopardizing the safety of the repair. IPOM-OG may be a suitable option in properly selected patients with small midline hernia defects.

## Introduction

Ventral hernia repair is a frequent procedure in abdominal wall surgery. There are many different surgical options, including mesh or suture repair, approach (open, laparoscopic, or robotic), mesh type, and fixation technique [1]. When it comes to laparoscopic intraperitoneal onlay mesh (IPOM) repair, one crucial point is the fixation method to prevent mesh migration and hernia recurrence [2]. The different fixation techniques are still debated in the surgical community, and a standard procedure has not yet been established [3]. At present, tacks (absorbable or non-absorbable) are most commonly used to secure the mesh in its intraperitoneal positioning [4]. In addition, trans-fascial sutures are sometimes used to reinforce mesh fixation. These two fixation methods pierce the abdominal wall, may cause nerve entrapment, and have been associated with significant acute postoperative pain, which in 10%-20% of patients persists after eight weeks [5]. Nevertheless, it is generally assumed that the stronger fixation (helical tackers, trans-fascial stitches) is the best option despite the risk of complications. However, this fact has also prompted the use of tissue sealants as a non-penetrative technique for mesh fixation [4,5].

Broadly, we can differentiate tissue sealants into two types: fibrin glue and cyanoacrylate (CA). CA shows a higher resistance bonding in comparison with fibrin sealant, making it very useful in surgical practice [2,4].

Many studies have reported on the safety of CA in laparoscopic inguinal hernioplasty [6]. Also, some studies in animals have proven that CA is equivalent to suture fixation in terms of histological response, adhesion formation, and recurrence [7,8], but little data are available on its use in ventral hernias, and to date, all clinical studies on laparoscopic ventral hernia have used a combination of CA and tacks or sutures for mesh fixation [9].

In the present study, we have analyzed the clinical use of CA as the single method (“only-glue”) for mesh fixation in laparoscopic repair of ventral hernias, with hernia recurrence as the primary outcome and postoperative pain and wound complications as secondary outcomes. Furthermore, we also report the operative findings in two patients who underwent another laparoscopic procedure shortly after IPOM-only glue (IPOM-OG) repair.

## Materials and methods

Patients

All consecutive patients with an indication for elective surgical repair of midline primary or recurrent ventral hernias were initially considered candidates for inclusion in this study. Patients deemed unsuitable for IPOM-OG due to hernia defects larger than 5 cm, midline ventral hernias with rectus abdominis divarication wider than 4 cm, or those with other simultaneous intra-abdominal procedures were excluded from the study. A senior surgeon (JPY) with sound experience in laparoscopic abdominal wall hernia surgery was present in all procedures, either as primary or assistant surgeon. All patients were fully informed about the procedure and signed the consent document.

A prospectively maintained database registered patients' demographics, American Society of Anesthesiologists (ASA) class, body mass index (BMI), comorbidity, and any previous abdominal surgery. Perioperative surgical reports registered the location and the widest diameter (centimeters) of the defect, closure of the defect, size of the prosthesis, duration of surgery, length of hospital stay (LOS), visual analog scale (VAS) postoperative pain at 7th day and one month postoperatively, wound complications within the first month postoperatively, and mid-term outcomes, including chronic pain, abdominal bulging and recurrence rate.

Physical follow-up examinations were periodically performed after seven days, 30 days, 90 days, and one year postoperatively, with hernia recurrence as the primary outcome. In case of doubt at physical examination, abdominal wall ultrasound was requested. Secondary outcomes were acute and chronic (> eight weeks) postoperative pain and abdominal bulging at the area of the hernia repair.

IPOM-OG technique

Patients receive antibiotic prophylaxis with intravenous cefazolin 2 g (ciprofloxacin 400 mg in allergic patients) before skin incision. After pneumoperitoneum by Veress needle at Palmer's point, with pressure established at 15 mmHg, a standard technique using three trocars (10, 5, and 5 mm) inserted at the left flank and a 30º 10 mm camera is used. First, an exhaustive examination of the abdomen is performed, the hernia is carefully manipulated and reduced, and urachal and round ligament fatty tissues above and below the hernia defect are detached from the parietal peritoneum to clear the landing area for the mesh. After completion of the dissection, the margins of the hernia defect are marked and measured at the skin with a percutaneous needle, and then a composite mesh is tailored to overlap at least 5 cm at all margins of the defect. Defects greater than 2 cm in diameter are closed before mesh placement (IPOM +) using intracorporeal running suture (V-Loc no. 0) or interrupted trans-fascial sutures (polypropylene 1), depending on the primary surgeon's preference.

**Figure 1:**
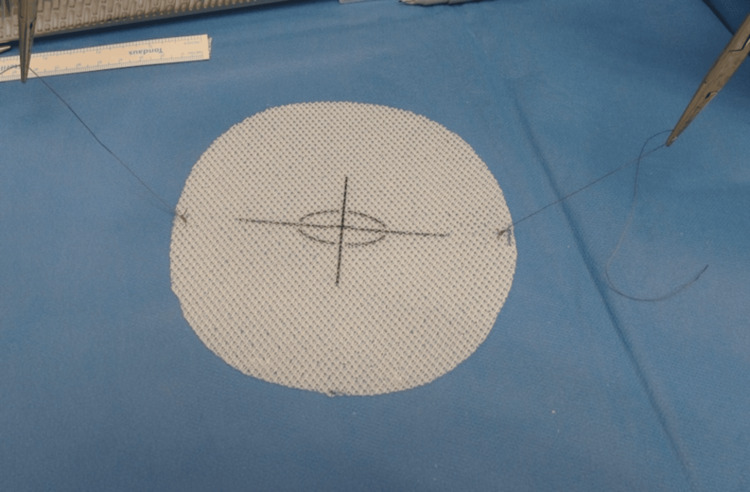
Vicryl 2-0 sutures placed at mesh margins to assist intracorporeal handling.

Before inserting the mesh inside the abdomen, two Vicryl 2-0 sutures are applied to both superior and inferior margins of the mesh, only used to aid in the intracorporeal handling of the mesh (Figure 1).

**Figure 2:**
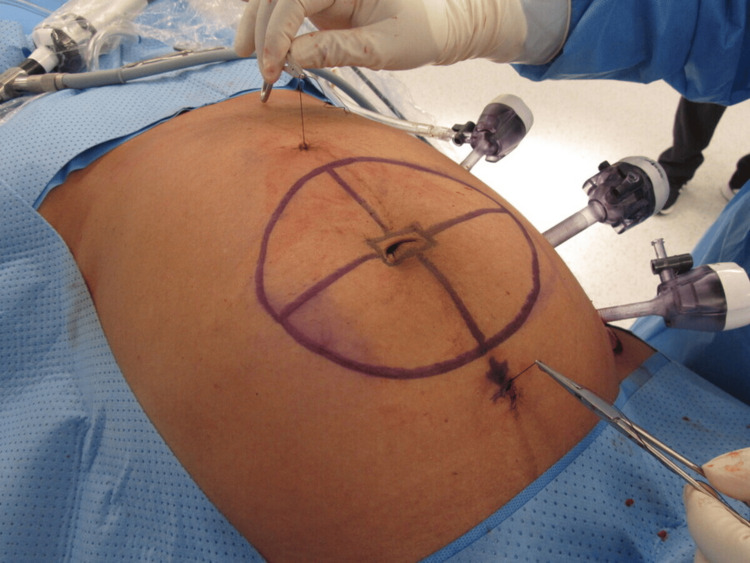
Percutaneous traction of exteriorized sutures positions the mesh against the peritoneum before glue application.

Once inside the abdomen, after exteriorizing these threads with a suture passer instrument, a percutaneous traction of both sutures through the skin helps for a proper positioning of the mesh against the peritoneal surface of the anterior abdominal wall prior to starting glue application (Figure 2).

**Figure 3:**
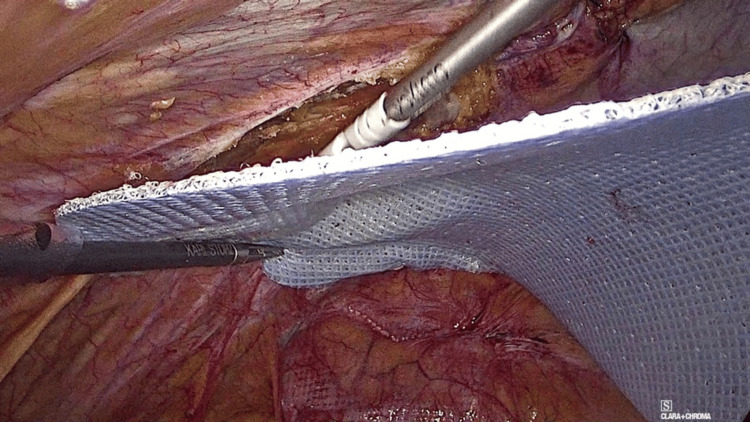
Articulating tip allows precise glue application between mesh and peritoneum, avoiding contact with intestines.

**Figure 4:**
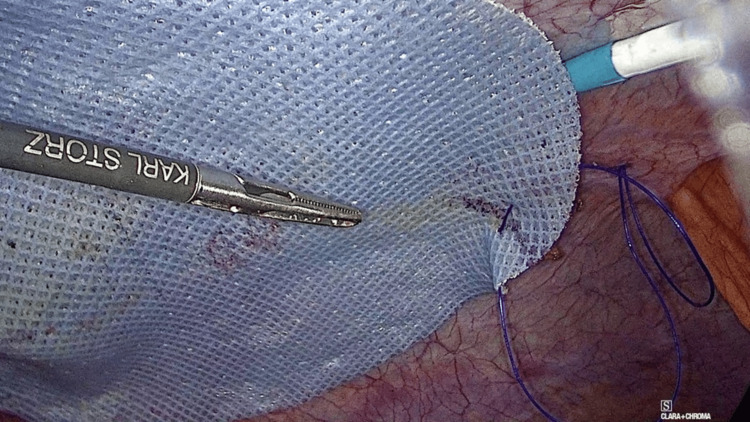
Mesh edges are curled to prevent glue leakage, and gentle upward pressure secures the mesh until the glue polymerizes.

Pneumoperitoneum pressure is reduced to 8-10 mmHg, the mesh is centered covering the defect, and then fixed using only Glubran 2® with GLUTACK® (GEM, Viareggio, Italy). Glubran 2® is a synthetic adhesive consisting of n-butyl-cyanoacrylate + methacryloxy sulfolane (NBCA + MS), which polymerizes quickly after about 60-90 seconds in contact with live wet tissue and creates a thin layer with a high tensile strength. GLUTACK® is a laparoscopic device for precise delivery of calibrated drops of GLUBRAN® 2 with each trigger activation, replicating other well-known mesh fixation devices. Interestingly, it is designed with an articulating tip for an optimal glue application. To avoid glue drops falling on the intestines, the glue application is always performed between the peritoneal surface and the mesh (Figure 3), curling up the borders of the prosthesis with a light pressure with a laparoscopic forceps onto the peritoneal wall for several seconds (Figure 4).

Once the fixation is completed, a revision of potential sources of bleeding in the abdominal cavity and trocar sites is carried out before emptying the pneumoperitoneum and removing the trocars.

Patients are transferred to the recovery room and are planned for discharge on a short-stay basis (less than 24 hours). An abdominal Velcro® girdle is immediately applied in the recovery room. Postoperative conventional analgesia, relative rest for one week, and follow-up in periodical outpatient consultation are recommended. Patients are advised to use the abdominal girdle for six to eight weeks.

## Results

A retrospective analysis of patients with midline ventral hernias who underwent IPOM-OG at Burjeel Medical City (Abu Dhabi, United Arab Emirates) from September 2022 to December 2023 has been carried out. Follow-up was available in 34 (85%) of the 40 patients who underwent IPOM-OG during the study period. Most patients were male (52%), with an average age of 42 years (Table 1).

**Table 1:** Demographic characteristics of the patients. The data are expressed as numbers (percentages), * except for median (interquartile range). BMI: body mass index; ASA: American Society of Anesthesiologists; COPD: chronic pulmonary obstructive disease.

Total patients	34
Gender	
Male	18 (52)
Female	16 (48)
Age*	42 (24-73)
BMI*	29 (21-35)
ASA classification	
I	2 (6)
II	18 (52)
III	14 (42)
IV	0 (0)
Comorbidity	
Diabetes	6 (17)
Anticoagulants	4 (12)
COPD	3 (9)
Types of previous operations	
Abdominal wall hernia	
Herniorrhaphy	3 (9)
Mesh repair	2 (6)
Laparoscopic cholecystectomy	1 (3)
Laparoscopic sleeve gastrectomy	1 (3)

The majority were primary umbilical hernias, with an average width of 3 cm. IPOM + was performed in 25 (74%) cases, most commonly using trans-fascial interrupted polypropylene sutures (median: 2 sutures, range: 1-4). Two types of composite mesh were used, i.e., Intra-Swing^TM^ (THT-Bio Science, Montpellier, France) or Symbotex^TM^ (Covidien, Trévoux, France), depending on the primary surgeon's preference. The average hernia-mesh size ratio was 1:16, the operative time was 64 (40-90) minutes, and there were no conversions to open surgery (Table 2).

**Table 2:** Hernia characteristics and operative data. The data are expressed as numbers (percentages), * except for median (interquartile range). EHS: European Hernia Society; IPOM: intraperitoneal onlay mesh.

Variables	n = 34
EHS classification	
M2 (epigastric)	7 (20)
M3 (umbilical)	24 (71)
M4 (infraumbilical)	3 (9)
Hernia width (cm):	
Average *	3 (1-5)
<2	14 (40)
2-5	20 (60)
Hernia type	
Primary	29 (85)
Recurrent	5 (15)
Closure of the defect	
Yes (IPOM +)	25 (74)
No (IPOM)	9 (26)
Size of mesh used, cm^2^*	200 (78-300)
Type of mesh used:	
THT Intra-Swing^TM^	28 (82)
Symbotex^TM^	6 (18)
Operative time*, minutes	64 (40-90)

There was no spillage of the glue over the intestine in any case. In one (3%) case, for unknown reasons, the CA did not work despite all technical steps being undertaken as described. Four (12%) patients developed port-site wound seroma, and two (6%) pseudo-hernias (seroma at the residual sac), confirmed by ultrasound, were successfully treated with fine-needle percutaneous aspiration. Postoperative VAS of pain in two different measures was low, with a majority of patients referring only mild pain, and no patient complained of chronic pain. After a median follow-up of 16 (12-26) months, no recurrences or abdominal bulging have been detected (Table 3).

**Table 3:** Postoperative outcomes. The data are expressed as numbers (percentages), * except for median (interquartile range).

Variables	n = 34
Surgical site occurrence	5 (15)
Port site wound seroma	4 (12)
Port site wound infection	1 (3)
Postoperative ileus	4 (12)
Postoperative ultrasound study	5 (15)
Normal	3 (9)
Pseudo-hernia	2 (6)
Postoperative pain (visual analog scale)*	
After one week	2 (1-3)
After one month	1 (0-2)
Length of stay* (days)	1.3 (1-3)
Chronic pain	0
Abdominal bulging	0
Recurrence	0
Follow-up (months)*	16 (12-26)

Two patients underwent another laparoscopic procedure (cholecystectomy) 14 and 90 days after IPOM-OG, respectively. In both cases, we could confirm that the mesh was adequately positioned without disruptions, ridges, wrinkles, bulges, or curled edges.

## Discussion

To the best of our knowledge, this is the first documented clinical study to investigate the use of glue as the only method for mesh fixation in laparoscopic IPOM repair of ventral hernias, and it is aimed to provide insights on a controversial topic, since the optimal method of mesh fixation in this type of surgical procedure is yet to be determined [1,5]. The most commonly used fixation techniques, i.e., tackers and sutures, are associated with significant complications such as acute and chronic pain due to nerve entrapment and bleeding [3,4]. This has prompted a search for mesh fixation using CA to reduce the risk of these complications. CA is probably the most frequently studied adhesive. It has demonstrated good results in inguinal hernia repair [2,8], but there are scarce data about its use in ventral hernia repair. In IPOM repair, CA use has been reported in animal studies, but unfortunately, there is a paucity of published clinical research. Until now we have found one study published in 2020 by Wilson [9], in which CA was the main method for mesh fixation in 137 laparoscopic IPOM repairs, but in all cases the surgeon used at least two to four transfascial stay sutures, and also some tacks in conjunction with CA for larger meshes.

Our premise in this study was to completely avoid the risks associated with tacks or transfascial sutures without jeopardizing the safety of the repair. With more than 24 years of experience in laparoscopic ventral hernia repair [10], in which we gradually diminished the number of tackers and started adding CA, we hypothesized that IPOM-OG could work well in selected small to medium-sized midline ventral hernias, especially if the hernia defect was closed. Certainly, in this study, we detected very low postoperative pain scores and no cases of chronic pain or hernia recurrence over a median follow-up period of 16 months.

Before starting our study, our main concern was related to the adequacy of CA as a valid fixation method. In terms of the fixation strength, several experimental studies have investigated the safety of IPOM repairs with CA, with conflicting results [7,8,11,12]. Recently, Végleur and Le Ruyet [11] conducted an ex vivo experiment that measured mesh bulging during an insufflation test, comparing the use of suture versus CA fixation in IPOM repair without defect closure. They found that, when compared to sutured meshes, glued ones had lower stiffness and higher bulging, and suggested that it could be due to the shallow CA fixation only to the peritoneum, whereas penetrating fixations such as sutures and tacks anchor the mesh deeper to the abdominal wall. However, although the experiment was designed to replicate in vivo conditions, the authors concluded that further investigations based on human models and closing the hernia defect should be conducted to address these conclusions.

In contrast, some other studies in animals have found that CA provides similar features to sutures in terms of hernia recurrence and histological response with tissue ingrowth [7,8]. An experimental study in a porcine abdominal wall defect model by Villalobos et al. [12] revealed collagen formation and the presence of myofibroblast cells and even mature bone tissue in areas where CA had been applied 12 weeks before. They also evaluated the tensile strength of the glued meshes, which resulted in values similar to those in previous studies using tacks. The authors concluded that laparoscopic mesh fixation with only CA might be feasible and safe in intraperitoneal incisional/ventral hernia repair, but again, it was suggested that closing the hernia defect would probably increase the safety of the repair. Nevertheless, there is a lack of consensus regarding the pros and cons of fascial defect closure, and some previous trials failed to show a statistically significant difference [13]. Although it looks uncertain for some authors [11], we found that when IPOM-OG was used without closure of the defect (always in hernias smaller than 2 cm), the results were invariably the same. In our opinion, various factors such as the smaller size of the hernias that were left unclosed, the absence of associated rectus divarication, and a relatively larger hernia-mesh size ratio can explain why we did not encounter any bulging or recurrence in this subset of patients.

From a technical standpoint, one criticism of CA use is the risk of severe adhesions when it comes into contact with the intestinal loops. Some strong precautions are required to prevent this from happening [14]. It has been suggested to cover the intestines with a gauze during CA application [4], but it has not been necessary in our study, as we always use coated meshes and the glue is always delivered on the parietal side of the mesh against the abdominal wall. The technique of using CA with the GLUTACK® device obviously differs from a tacking technique, but we found the learning curve to be almost inexistent. Traction of the percutaneous sutures not only helps in achieving a centered position of the mesh to the hernia defect, but also aligns the mesh with the inner abdominal wall and prevents CA spillage during its delivery. With this quite simple technique, we have not encountered any case of CA spillage to intra-abdominal organs. Of note, we agree with Villalobos et al. [12] that it is crucial to apply controlled drops of CA, delivering a single shot in each fixating point, because an excess of glue can saturate the mesh-peritoneum interface, leading to a suboptimal mesh fixation due to the formation of non-absorbed rigid residues.

There is growing evidence that CA reduces the risk of postoperative complications, such as pain and bleeding [15-18]. Apart from the fixation method and the closure of the defect, it is well known that postoperative pain after IPOM varies due to multiple factors, such as individual differences in pain perception, abdominal wall compliance, BMI, or type of physical activity. Furthermore, some surgeons may opt not to close the defect to prevent excessive tension within the abdominal wall, which may result in possible pain [11]. Although the impact of these factors cannot be overlooked, our results show a very low VAS score after IPOM-OG, and it can be assumed that the postoperative pain is mainly secondary to the closure of the defect, rather than the effect of CA to secure the mesh.

**Figure 5:**
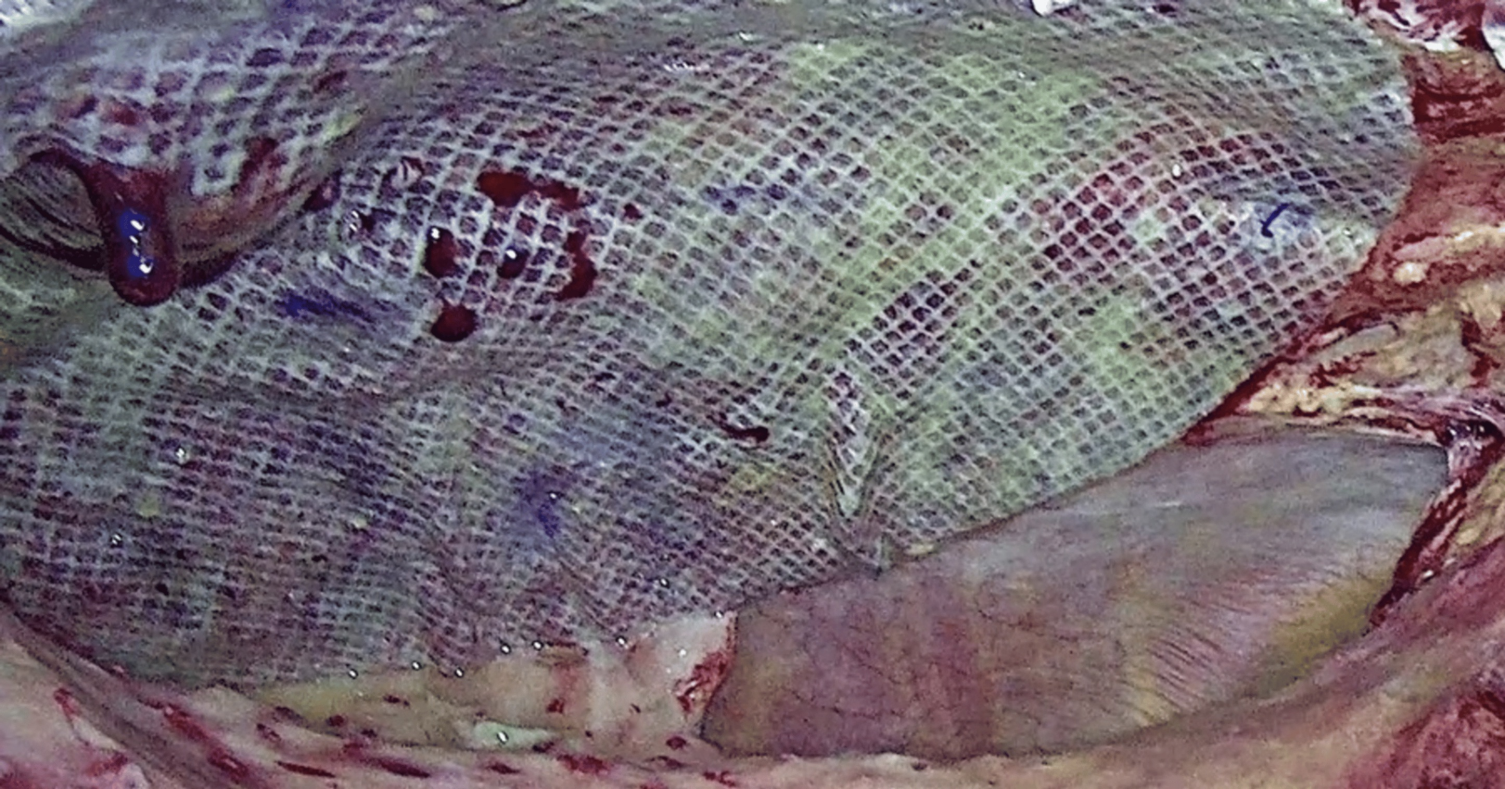
Laparoscopic view shows thin, avascular, easily removable adhesions and solid mesh integration after IPOM-OG. IPOM-OG:

**Figure 6:**
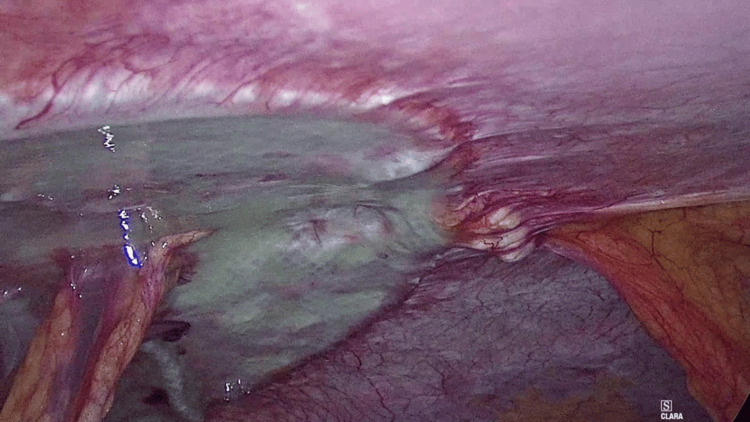
Thin, avascular, and uniformly distributed adhesions observed on the mesh surface, easily separated during laparoscopy.

Finally, a main concern with tack fixation is the occurrence of adhesions that can lead to life-threatening complications. Intestinal obstructions and perforations due to exposed titanium tacks have been observed [19]. Several recent studies have found mild inflammatory tissue reaction and good mesh integration after CA fixation [2,4,8,9,12], and consequently, it can be argued that adhesions may be reduced by the use of CA instead of tacks. However, unless a reoperation is carried out, it seems difficult to prove a significant reduction in the formation of intraperitoneal adhesions with the use of CA instead of tacks in a clinical scenario. In this sense, there were no bowel obstruction events (postoperative colic abdominal pain or intestinal obstruction) in our study. But of interest, we had the opportunity to directly assess the adhesion formation after IPOM-OG. As we previously mentioned, two patients underwent laparoscopic cholecystectomy due to acute cholecystitis and recurrent biliary colic 14 and 90 days after IPOM-OG, respectively. In both cases, we found thin intraperitoneal adhesions, without vessels, easily removable, and distributed homogeneously along the surface of the mesh. We could also confirm the solid integration of both meshes, even though in the first case the mesh had been placed only two weeks before (Figures 5, 6).

There are several limitations in this study, such as the small number of cases and a relatively short follow-up. While we assessed the recurrence rate as the primary outcome, periodical follow-up is warranted to comprehensively evaluate the long-term clinical value of the IPOM-OG technique in ventral hernia repair. Also, there is a lack of comparison to a group with tack fixation, and also a criticism could be raised for some selection bias (type of hernia and patient's characteristics) as responsible for the low VAS score and recurrence rate.

## Conclusions

In summary, this study adds further evidence about the efficacy and safety of CA as a sole fixation method in IPOM repair of midline ventral hernias. IPOM-OG is associated with a low postoperative pain and hernia recurrence, and avoids adverse events such as abdominal wall bleeding, nerve injury, and chronic pain linked to conventional tacks and suturing techniques. The IPOM-OG repair has almost no learning curve and is simple to standardize. Our experience supports the idea that IPOM-OG may be of choice in properly selected patients with small midline hernia defects. Although these results are encouraging, further prospective studies comparing CA with or without supplementary tactics are needed.
